# Global prevalence and risk factors of equine infectious anemia: A systematic review and meta-analysis

**DOI:** 10.14202/vetworld.2025.1440-1451

**Published:** 2025-06-06

**Authors:** Lintang Winantya Firdausy, Faisal Fikri, Arya Pradana Wicaksono, Hakan Çalışkan, Muhammad Thohawi Elziyad Purnama

**Affiliations:** 1 Division of Veterinary Medicine, Department of Health and Life Sciences, Faculty of Health, Medicine, and Life Sciences, Universitas Airlangga, Banyuwangi, East Java, 68425, Indonesia; 2 Research Group of Animal Biomedical and Conservation, Faculty of Health, Medicine, and Life Sciences, Universitas Airlangga, Banyuwangi, East Java, 68425, Indonesia; 3 Animal Health Division, Indonesian Horse Veterinarian Association, Surabaya, East Java, 60115, Indonesia; 4 Department of Biology, Faculty of Science, Eskisehir Osmangazi University, Eskisehir, 26040, Turkey; 5 Department of Biology, Graduate School of Natural and Applied Sciences, Eskisehir Osmangazi University, Eskisehir, 26040, Turkey

**Keywords:** agar gel immunodiffusion, enzyme-linked immunosorbent assay, Equidae, equine infectious anemia, global prevalence, infectious disease, lentivirus, meta-analysis, seroepidemiology

## Abstract

**Background and Aim:**

Equine infectious anemia (EIA) is a lentiviral disease affecting members of the Equidae family, with global distribution and significant implications for animal health and biosecurity. Despite numerous individual reports, a comprehensive synthesis of its global prevalence and risk factors remains lacking. This study aimed to conduct a systematic review and meta-analysis to estimate the global prevalence of EIA, identify diagnostic trends, and evaluate factors associated with heterogeneity across studies.

**Materials and Methods:**

A systematic search was conducted in six major databases (PubMed, Scopus, Web of Science, ScienceDirect, Cochrane Library, and ProQuest), yielding 312 records. After Preferred Reporting Items for Systematic Reviews and Meta-Analyses-guided screening, 29 eligible studies published between 1975 and 2024 were included in the study. Meta-analysis was performed using R Studio (version 4.4.2) employing a random-effects model. Subgroup analyses and meta-regression were conducted to explore heterogeneity across host species, continent, diagnostic method, and study period. Publication bias was assessed through funnel plots and Egger’s test.

**Results:**

The global pooled prevalence of EIA was estimated at 20.97% (95% confidence interval [CI]: 11.08–30.85), with substantial heterogeneity (I^2^ = 99.3%). South America reported the highest regional prevalence (27.21%), while horses showed the greatest susceptibility among Equidae (25.40%). Diagnostic methods varied, with agar gel immunodiffusion being the most commonly used (18.62% prevalence detection). A declining trend in prevalence (2.19%–28.70%) was noted from 2015 to 2022. No significant publication bias was detected. Meta-regression revealed that climate and study period partially explained the heterogeneity.

**Conclusion:**

This study highlights the substantial global burden and diagnostic variability of EIA, emphasizing the need for enhanced surveillance in endemic areas, standardized diagnostic protocols, and strengthened quarantine practices. Expanding serological monitoring in underrepresented regions and integrating climatic and ecological data into control strategies are vital for mitigating EIA transmission risks.

## INTRODUCTION

Equine infectious anemia (EIA), also referred to as swamp fever, is a globally distributed disease caused by a Lentivirus within the *Orthoretrovirinae* subfamily of the *Retroviridae* family, which primarily infects equids – namely, horses, mules, donkeys, and zebras [1]. Clinically, EIA manifests in three distinct forms: acute, chronic, and inapparent (asymptomatic) infections. The severity and clinical presentation of the disease vary considerably depending on host factors and the virulence of the viral strain, with elevated viremia levels typically observed during early or severe infection. The chronic form is most frequently encountered and is characterized by thrombocytopenia, anemia, dependent edema, pyrexia, lethargy, anorexia, progressive weight loss, and, less commonly, neurological manifestations such as ataxia and encephalitis [2]. In contrast, the acute form may present with high fever, anorexia, hemolytic anemia, and edema, potentially culminating in death [3]. Animals that survive the acute phase generally transition to either a chronic or inapparent state of infection. Due to its often subtle clinical signs, the acute form is frequently underdiagnosed, contributing to persistent transmission cycles [4].

Natural transmission of EIA virus (EIAV) occurs predominantly through hematophagous *Tabanidae* species (horseflies), which act as mechanical vectors by transferring infected blood between hosts during interrupted feeding episodes. Iatrogenic routes, including the reuse of contaminated syringes, needles, or intravenous equipment, also play a critical role in disease spread [5]. Although the virus does not replicate within insect vectors, it can remain viable on the mouthparts of biting flies such as horseflies and stable flies (*Stomoxys calcitrans*) for several hours post-exposure, facilitating indirect transmission [6]. Infected equines serve as lifelong reservoirs of EIAV and pose a consistent transmission threat, irrespective of their clinical status. To date, no effective vaccine or curative treatment for EIA exists, rendering infected animals a perpetual epidemiological risk [7].

The global distribution of EIA includes endemic regions and areas experiencing sporadic outbreaks. While endemicity has been reported in parts of North and South America, Asia, and Eastern Europe, regions such as the European Union have largely contained the disease to isolated outbreaks through rigorous surveillance and control measures [8].

Despite the global relevance of EIA, the epidemiological landscape remains inadequately characterized across many geographic regions, particularly in underrepresented areas such as parts of Africa, Oceania, and Central Asia. While multiple national and regional surveillance studies have assessed EIA prevalence using various serological and molecular diagnostics, these investigations are frequently limited by heterogeneous study designs, inconsistent reporting standards, small sample sizes, and variable diagnostic sensitivity. Moreover, previous systematic reviews addressing EIA have been either narrowly focused on specific host species or restricted to limited geographical scopes, failing to provide a comprehensive and updated global synthesis. There remains a critical need to quantify the pooled global burden of EIA, examine temporal trends over recent decades, and elucidate the influence of diagnostic methods, host species, and ecological or regional factors on prevalence variability. In addition, the lack of standardized analytical integration of climatic, biological, and methodological covariates has limited our understanding of the complex drivers of EIA distribution.

Therefore, this study aimed to conduct a comprehensive systematic review and meta-analysis to estimate the global pooled prevalence of EIA across members of the Equidae family from 1975 to 2024. Specifically, this study sought to (i) assess the temporal trends in EIA occurrence, (ii) evaluate the impact of host species, geographical region, and diagnostic technique on prevalence estimates, and (iii) investigate potential sources of heterogeneity through subgroup analysis and meta-regression. By synthesizing data from multiple regions and standardizing prevalence estimates, this review provides evidence-based insights to inform targeted surveillance strategies, refine diagnostic protocols, and guide risk-based disease control policies on a global scale.

## MATERIALS AND METHODS

### Ethical approval

**Figure 1:**
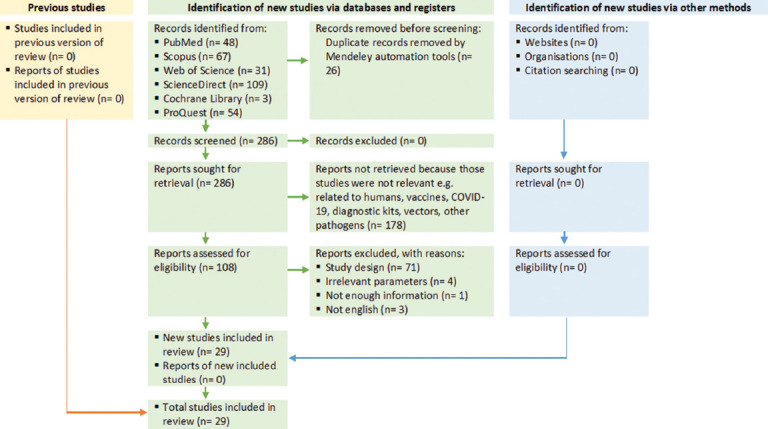
Preferred Reporting Items for Systematic Reviews and Meta-Analyses flow diagram of the study selection process.

This systematic review followed the 2020 Preferred Reporting Items for Systematic Reviews and Meta-Analyses (PRISMA) guidelines [9], including the use of a PRISMA flow diagram to detail the study selection process (Figure 1). As this review did not involve live animals or clinical interventions, formal ethical approval was not required.

### Registration

The literature search protocol is registered with the Open Science Framework. DOI: 10.17605/OSF.IO/S7WF9 (https://osf.io/s7wf9/).

### Study period and locations

All phases of literature screening, data extraction, statistical analysis, and visualization were conducted between August 20, 2024, and January 17, 2025, at Eskisehir Osmangazi University, Turkey, and Universitas Airlangga, Indonesia.

### Search strategy and study selection

A comprehensive search was performed across six major databases – PubMed, Scopus, Web of Science, ScienceDirect, Cochrane Library, and ProQuest – to identify studies reporting the global prevalence or incidence of EIA. The search strategy adhered to the Peer Review of Electronic Search Strategies 2015 guidelines and was reviewed by a veterinary epidemiology expert.

Four reviewers (LWF, FF, HÇ, and MTEP) independently screened all titles and abstracts, followed by full-text evaluations. Discrepancies were resolved through consensus with a fifth reviewer (APW). The study design adhered to the PICOS framework (Table 1) [10–38] for structured selection and extraction:

**Table 1:** Searching strategy based on the PICOS method.

PICOS items	PICOS	Keywords
Population	Equine infectious anemia	“equine infectious anemia” [MeSH Terms] OR “horsemen” [Title/Abstract] OR “swamp fever” [Title/Abstract]
Interventi on	Not applied	Not applied
Comparison	Not applied	Not applied
Outcomes	Primary outcome: outbreak Secondary outcomes: incidence and prevalence	“outbreak” [Title/Abstract] OR “incidence” [Title/Abstract] OR “prevalence” [Title/Abstract]
Study Design	Observational studies	“outbreak” [Title/Abstract] OR “incidence” [Title/Abstract] OR “prevalence” [Title/Abstract]

- Population (P): Equidae (horses, donkeys, mules, and zebras) across all ages, breeds, and sexes with confirmed natural EIA infection
- Intervention (I): Not applicable; the study focused on observational data regarding seroprevalence and incidence
- Comparison (C): Not applicable; no control group was necessary for summarizing prevalence estimates
- Outcome (O): The primary outcome was EIA prevalence; secondary outcomes included stratified prevalence by continent, host, diagnostic test, and temporal trends
- Study Design (S): Observational designs including cross-sectional studies, retrospective reports, and descriptive epidemiology. Studies had to report both the number of EIA-positive cases and total sample size. Reviews, experimental infections, and studies lacking prevalence data were excluded from the study.

Keywords such as “equine infectious anemia,” “prevalence,” and “incidence” were employed. Advanced Boolean operators and Medical Subject Headings (MeSH) terms ensured comprehensive retrieval. For instance: #1 “equine infectious anemia” [MeSH Terms] OR “horsemen” [Title/Abstract] OR “swamp fever” [Title/Abstract]; #2 “outbreak” OR “incidence” OR “prevalence” [Title/Abstract].

### Eligibility criteria

Reference management and duplicate removal were performed using Mendeley software version 1.19.5 (Mendeley Ltd., Elsevier, Netherlands), with exports in .txt and .csv formats. Titles and abstracts were reviewed for relevance, with no language restriction during initial screening; however, only English-language articles were included in the final analysis. Exclusions were applied to non-English articles, reviews, experimental studies, papers lacking full texts, or those not reporting key epidemiological variables.

Studies published between 1975 and 2024 were considered. Methodological rigor was evaluated using the Joanna Briggs Institute (JBI) critical appraisal checklist. Articles were scored across key domains: clarity of objectives, stated location and time frame, sample subgrouping, sampling methods, and diagnostic approaches (Table 2). All inclusion decisions were documented following PRISMA guidelines (Figure 1).

**Table 2:** Study quality assessment showing the number of included studies in each category that adopted the Joanna Briggs Institute critical appraisal checklist.

Items	Total number of included studies			

	Yes	No	Unclear	Not applicable
Was the research objective clearly described and stated?	100	0	0	N/A
Was the study period and location clearly stated?	93.1	0	6.89	N/A
Was the sample categorized into different subgroups?	100	0	0	N/A
Was the sampling method described in detail?	79.31	10.34	10.34	N/A
Was the diagnostic technique clearly identified?	100	0	0	N/A
Maximum	100	20	20	N/A
Minimum	60	0	0	N/A
Median	80	10	10	N/A

N/A=Data not available

### Data extraction

Extracted variables included authorship, publication year, study location and duration, number of cases, total sample size, prevalence and mortality rates, host species, sample types, and diagnostic methods. Data discrepancies were resolved by consensus among the authors. All extracted information was compiled using Microsoft Excel (Microsoft Corp., Redmond, WA, USA), and spatial data visualizations were generated with QGIS software version 3.22.8 (QGIS Association; Białowieża. https://qgis.org/).

### Statistical analysis

All meta-analytical computations were performed in R Studio (version 4.4.2, Posit PBC, USA) using the “meta” package. Prevalence data were treated as continuous, while event counts and sample sizes were treated as dichotomous. Forest plots based on log odds ratios were used to present cumulative prevalence estimates. Meta-regression was used to examine temporal trends and subgroup effects. Scatter plots with 95% confidence and prediction intervals illustrated the results.

A random-effects model incorporating Tau-squared (τ^2^) was used to account for between-study variance. Heterogeneity was assessed using the I^2^ statistic, where values >50% and p < 0.05 indicated substantial heterogeneity. Subgroup analyses were stratified by host species, diagnostic methods, study periods, and geographic regions. Publication bias was assessed through funnel plot asymmetry and Egger’s regression test.

## RESULTS

### Identification and selection of studies

A total of 312 articles were initially retrieved from six electronic databases: PubMed (48), Scopus (67), Web of Science (31), ScienceDirect (109), Cochrane Library (3), and ProQuest (54). Following the removal of duplicates, 286 articles were screened for relevance based on title and abstract. Of these, 178 articles were excluded for reasons including non-equine subject focus, vaccine studies, COVID-19-related content, or unrelated pathogens. An additional 79 articles were excluded due to being non-English (n = 3), employing unsuitable study designs (n = 71), lacking essential epidemiological data (n = 1), or reporting irrelevant parameters (n = 4). The detailed process of identification, screening, and eligibility assessment is summarized in the PRISMA flow diagram (Figure 1).

### Characteristics of included studies

Ultimately, 29 studies met the inclusion criteria and were incorporated into the quantitative meta-analysis. These studies spanned 16 countries across five continents, comprising 17 from Europe, 5 from Asia, 13 from South America, 5 from North America, and 1 from Australia. Collectively, the included studies encompassed 27,909 samples: 2,879 from Europe, 5,844 from Asia, 15,778 from South America, 3,004 from North America, and 451 from Australia.

**Figure 2:**
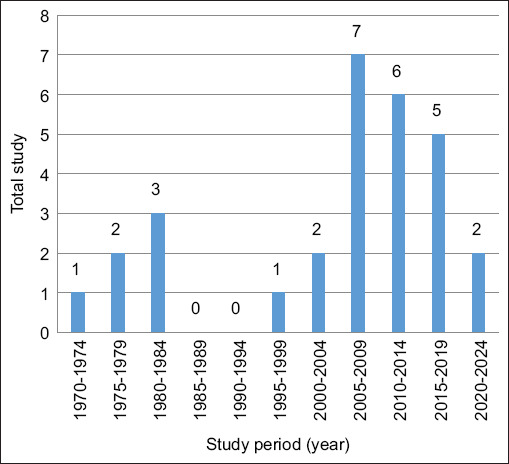
Distribution of studies relevant to equine infectious anemia since 1975.

Host distribution included 28 studies on horses (n = 26,293), 4 on donkeys (n = 1,500), and 2 on mules (n = 116). The diagnostic methods applied across the studies included agar gel immunodiffusion (AGID) (n = 25,421), enzyme-linked immunosorbent assay (ELISA) (n = 6,474), polymerase chain reaction (PCR) (n = 400), and combination testing methods (n = 342) (Table 3). The earliest surveillance data included in this analysis dated back to a 1975 study from the United States. Surveillance frequency peaked between 2005 and 2009, followed by a gradual decline, with only two studies conducted between 2020 and 2024 (Figure 2).

**Table 3:** Characteristics of the included studies.

Study period	Country	Events	Sample size	Prevalence (%)	Mortality (%)	Host	Sample type	Test	References
1980	Guyana	136	226	60.18	N/A	Horse	Serum	AGID	[10]
2014	Brazil	664	3858	17.2	N/A	Horse	Serum	AGID	[11]
2002–2004	Brazil	284	9031	3.14	N/A	Horse	Serum	AGID	[12]
2009	Brazil	13	47	27.7	N/A	Mule	Serum	AGID	[13]
		121	500	24.2	N/A	Horse	Serum	AGID	
2010	Belgium	6	95	6.32	N/A	Horse	Serum	AGID	[14]
2006–2009	Italy	70	400	17.5	N/A	Horse	Serum	PCR	[15]
		53	400	13.25	N/A	Horse	Serum	AGID	
2006	Ireland	18	32	56.25	N/A	Horse	Serum	AGID, ELISA	[16]
2008, 2015	Brazil	23	23	100	N/A	Horse	Serum	ELISA	[17]
1980	USA	6	8	75	N/A	Horse	Serum	AGID	[18]
2009	France	16	250	6.4	N/A	Horse	Serum	AGID, PCR	[19]
2018	Argentina	20	20	100	N/A	Horse	Serum	AGID	[20]
2009–2012	Canada	72	514	14	N/A	Horse	Serum	AGID	[21]
1975	USA	94	1398	6.7	N/A	Horse	Serum	AGID	[22]
2006	Ireland	35	38	92.11	N/A	Horse	Serum	AGID, ELISA, PCR	[23]
2006	Ireland	13	22	59.09	23.08	Horse	Serum	AGID, ELISA, PCR	[24]
1982	Guyana	110	678	16.22	11.1	Horse	Serum	AGID	[25]
2009–2011	Brazil	6	367	1.6	N/A	Donkey	Serum	AGID	[26]
		12	367	3.3	N/A	Donkey	Serum	ELISA	
		53	367	14.4	N/A	Donkey	Serum	ELISA	
2020–2021	Brazil	14	1170	1.2	N/A	Horse	Serum	AGID	[27]
2006	Ireland	4	5	80	N/A	Horse	Serum	AGID	[28]
2018–2020	Brazil	2	28	7.14	N/A	Horse	Serum	AGID	[29]
2016	Mongolia	11	776	1.42	N/A	Horse	Serum	AGID	[30]
1971–1973	Australia	34	451	7.54	N/A	Horse	Serum	AGID	[31]
2003–2004	Türkiye	0	408	0	N/A	Horse	Serum	AGID	[32]
		0	69	0	N/A	Mule	Serum	AGID	
		0	154	0	N/A	Donkey	Serum	AGID	
2006–2015	Saint Kitts and Nevis, the	0	140	0	N/A	Horse	Serum	ELISA	[33]
		0	40	0	N/A	Donkey	Serum	ELISA	
2011–2013	Italy	0	555	0	N/A	Horse	Serum	ELISA	[34]
1978	Oman	0	86	0	N/A	Horse	Serum	AGID	[35]
2017–2019	Saudi Arabia	0	4523	0	N/A	Horse	Serum	ELISA	[36]
		0	205	0	N/A	Donkey	Serum	ELISA	
2011	Jordan	0	254	0	N/A	Horse	Serum	ELISA	[37]
1997–1998	Türkiye	0	404	0	N/A	Horse	Serum	AGID	[38]

N/A=Data not available, AGID=Agar gel immunodiffusion, ELISA=Enzyme-linked immunosorbent assay, PCR=Polymerase chain reaction.

**Figure 3:**
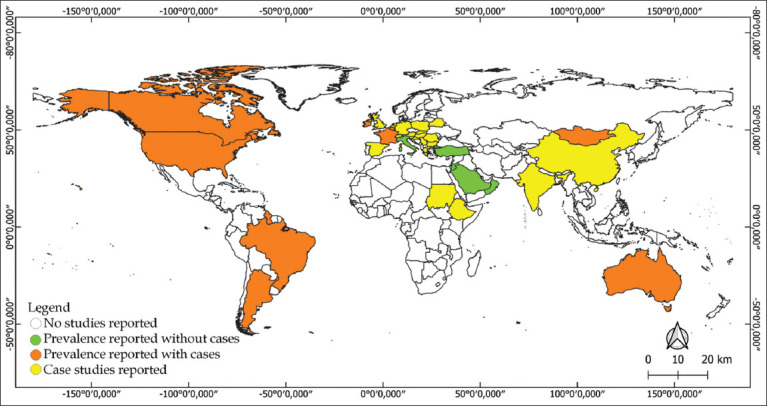
Distribution of equine infectious anemia worldwide based on studies conducted since 1975. Distribution of study reports visualized using QGIS v3.22.8.

A geospatial visualization (Figure 3) was used to map the distribution of EIA reports. Countries with documented surveillance and positive cases are marked in orange [10–31]. Countries with surveillance but no reported positive cases are marked in green [32–38]. Yellow indicates nations with isolated reports and presumed insufficient surveillance [39–44]. White regions represent countries without reported EIA data but remain at risk due to proximity to endemic zones.

### Quality assessment of included studies

Study quality was assessed using the JBI critical appraisal checklist. Among the 29 included studies, methodological quality was rated as follows: maximum score of 100%, minimum score of 60%, and a median score of 80%. Sub-items receiving a “No” or “Unclear” designation had maximum values of 20%, minimum values of 0%, and a median of 10% (Table 2).

### Pooled global prevalence of EIA

**Figure 4:**
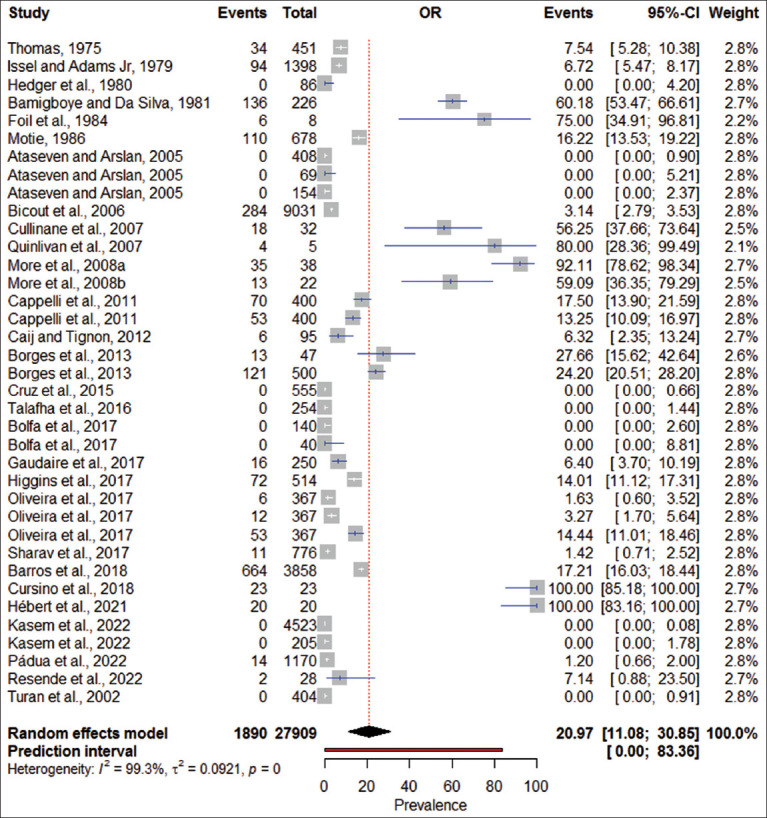
Forest plot of cumulative prevalence and 95% confidence intervals of equine infectious anemia across studies.

The meta-analysis estimated a global pooled prevalence of 20.97% (95% confidence interval [CI]: 11.08–30.85) for EIA among Equidae. The analysis revealed significant heterogeneity (I^2^ = 99.30%, τ^2^ = 0.0921, p < 0.0001). Prevalence estimates across surveillance-implementing nations between 1975 and 2022 ranged from 0.00% to 83.36%. A declining fluctuation trend was observed during 2015–2022, with prevalence estimates varying from 2.19% to 28.70% (Figure 4).

### Subgroup meta-analysis

Subgroup analyses identified statistically significant differences in EIA prevalence based on continent (p < 0.0001), study period (p < 0.0001), host species (p = 0.0028), and diagnostic method (p < 0.0001). The highest estimated prevalence (48.58%, 95% CI: 13.80–83.37) occurred during 1980–1984. Regionally, South America showed the highest prevalence (27.21%, 95% CI: 5.29–49.13). Horses exhibited the greatest susceptibility (25.40%, 95% CI: 13.21–37.69), followed by mules and donkeys.

Among diagnostic tools, combined AGID, ELISA, and PCR methods demonstrated the highest detection sensitivity (76.97%, 95% CI: 44.73–100.00), whereas AGID alone – most frequently used – yielded an accuracy of 18.62% (95% CI: 7.51–29.72) (Table 4).

**Table 4:** Overall pooled prevalence of equine infectious anemia and subgroup meta-analysis.

Categories	Total studies or subgroups	Prevalence (%)		Heterogeneity			p-value for subgroup difference
	
		Estimate	95% CI	I^2^ (%)	τ^2^	p-value	
Overall	29	20.97	11.08–30.85	99.30	0.0921	0.000	
Study period							
• 1970–1974	1	N/A	N/A	N/A	N/A	N/A	<0.0001
• 1975–1979	2	3.38	0.00–9.97	97.60	0.0022	<0.0001	
• 1980–1984	3	48.58	13.80–83.37	98.80	0.0874	<0.0001	
• 1995–1999	1	N/A	N/A	N/A	N/A	N/A	
• 2000–2004	4	0.86	0.00–2.50	98.20	0.0003	<0.0001	
• 2005–2009	9	40.27	20.15–60.40	98.00	0.0892	<0.0001	
• 2010–2014	8	7.03	2.07–11.99	99.20	0.0050	<0.0001	
• 2015–2019	7	28.70	0.00–64.68	99.70	0.2357	0.0000	
• 2020–2024	2	2.19	0.00–6.63	32.70	0.0006	0.2228	
Continent							
• Australia	1	N/A	N/A	N/A	N/A	N/A	<0.0001
• North America	7	22.77	2.02–43.52	98.80	0.0755	<0.0001	
• Asia	5	0.24	0.00–0.75	64.10	<0.0001	0.0250	
• Europe	13	23.91	6.23–41.59	98.30	0.1022	<0.0001	
• South America	11	27.21	5.29–49.13	99.60	0.1367	0.0000	
Host							
• Horse	29	25.40	13.21–37.69	99.40	0.1096	0.0000	0.0028
• Mule	2	13.08	0.00–40.14	94.30	0.0361	<0.0001	
• Donkey	6	3.08	0.00–7.36	93.10	0.0027	<0.0001	
Test							
• AGID	23	18.62	7.51–29.72	99.20	0.0716	0.0000	
• AGID, ELISA	1	N/A	N/A	N/A	N/A	N/A	
• AGID, ELISA, PCR	2	76.97	44.73–100.00	88.20	0.0480	0.0037	
• PCR	1	N/A	N/A	N/A	N/A	N/A	
• ELISA	9	13.01	0.00–34.41	99.40	0.1072	<0.0001	
• AGID, PCR	1	N/A	N/A	N/A	N/A	N/A	<0.0001

95% CI=95% confidence intervals, I^2^=The primary index for reporting heterogeneity, τ^2^=Tau-squared focuses on the variability of true effect sizes, N/A=Data not available, AGID=Agar gel immunodiffusion, ELISA=Enzyme-linked immunosorbent assay, PCR=Polymerase chain reaction

### Meta-regression and publication bias analysis

**Figure 5:**
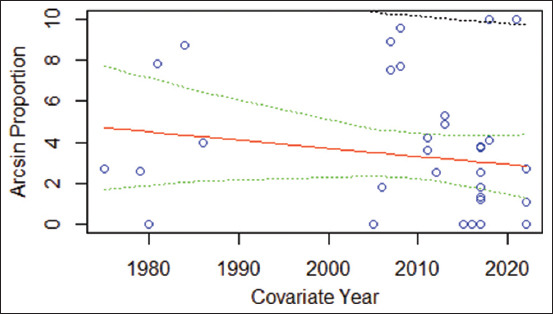
Scatter plot of the meta-regression analysis to evaluate trends in the prevalence of equine infectious anemia since 1975. The red line (---) represents the regression line, the green line (---) represents the 95% confidence interval, and the black line (---) represents the 95% prediction interval.

Meta-regression revealed no significant correlation between study year and EIA prevalence (−0.0401x + 83.887, 95% CI: −4.5107–7.1330, R^2^ = 0.0272, F = 0.2661) (Figure 5). Cumulative meta-analysis showed a peak in EIA prevalence during 1980–1984 (48.58%, 95% CI: 13.80–83.37), a marked decline to 0.86% in 2000–2004, followed by a sharp rise to 40.27% in 2005–2009, and a subsequent decrease from 2010 to 2024 (range: 2.19–28.70%).

**Figure 6:**
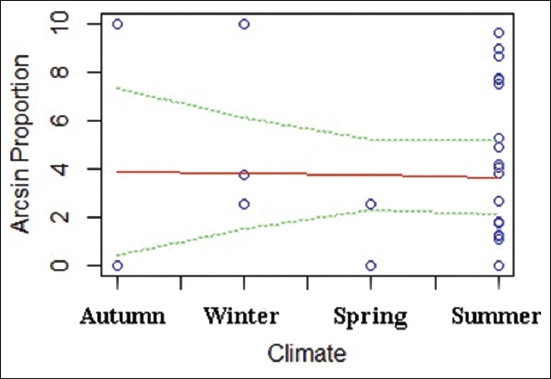
Scatter plot of the meta-regression analysis to evaluate the correlation between climate and the prevalence of equine infectious anemia. The red line (---) represents the regression line, the green line (---) represents the 95% confidence interval, and the black line (---) represents the 95% prediction interval.

Climatic conditions were also analyzed as moderators. A weak correlation was identified between climate and EIA prevalence (−0.07013x + 3.93733, 95% CI: −3.867–6.203, R² = 0.0004322, F = 0.01211) (Figure 6), with higher prevalence during summer months and lowest levels reported in autumn and spring.

**Figure 7:**
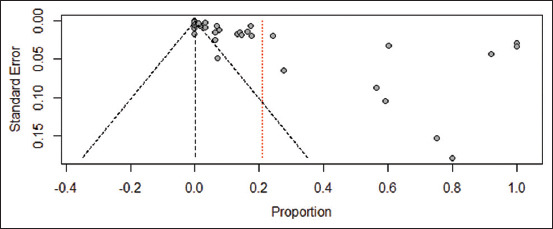
Funnel plot of standard error to evaluate publication bias in the prevalence of equine infectious anemia across studies.

Funnel plots revealed no visible asymmetry, and Egger’s regression test indicated no significant publication bias (p = 0.3769) (Figure 7). Of the included studies, 13 were rated as high quality, while 16 were assessed as having moderate methodological quality.

## DISCUSSION

This systematic review and meta-analysis aimed to estimate the global prevalence of EIAV in Equidae and to identify the contributing epidemiological, ecological, and methodological factors that explain its geographic and temporal variability. The study also examined diagnostic tools and risk determinants associated with EIA transmission and assessed the robustness of the existing surveillance data across regions.

### Summary of findings and transmission dynamics

This meta-analysis synthesized data from 29 studies comprising 27,909 samples from horses, donkeys, and mules across 16 countries. The global pooled prevalence of EIA was estimated at 20.97% (95% CI: 11.08–30.85), with notable variability between regions. Although moderate at the global level, the wide prediction interval suggests the risk of severe localized outbreaks.

The mechanical transmission of EIAV is primarily mediated by hematophagous vectors, notably *Tabanus* spp., *Chrysops* spp., and *S. calcitrans*. These vectors facilitate virus transfer between infected and susceptible hosts during interrupted feeding events. However, iatrogenic factors – such as contaminated medical equipment and blood-derived products – remain the most significant modes of transmission in certain regions [6]. Seasonal patterns, particularly during summer and late spring, coincide with peak vector activity, reinforcing the importance of ecological context in EIA epidemiology [45].

EIAV, one of the smallest lentiviruses (~8.2 kb), encodes structural genes (*gag*, *pol*, *env*) and accessory genes (*Tat*, *Rev*, *S2*) responsible for immune evasion and pathogenesis [46].

### Geographic distribution and surveillance patterns

Historical and current surveillance data revealed that South America exhibited the highest prevalence (27.21%), followed by Europe (23.91%) and North America (22.77%). Exceptional seroprevalence rates were observed in Guyana (60.18%) [10], Brazil and Argentina (100%) [17, 20], and Ireland (92.11%) [23]. Conversely, countries such as Italy, Türkiye, Saudi Arabia, and Jordan have recently reported either declining or absent EIA cases, suggesting effective control strategies or limited virus circulation.

### Host-specific susceptibility and exposure risks

The analysis showed host-specific differences in EIA prevalence. Horses exhibited the highest prevalence (25.40%, 95% CI: 13.21–37.69), followed by mules (13.08%) and donkeys (3.08%). Although historically considered lower-risk, mules are often used in labor-intensive tasks in remote areas with minimal veterinary oversight. These animals are frequently exposed to stress and competent vector populations, increasing the likelihood of viremia reactivation. Surveillance among donkeys remains sparse and potentially underestimates true prevalence due to small sample sizes and low population densities in many studies [47]. The use of mules in rural industries and insufficient biosecurity practices complicate control efforts in such marginal zones. Moreover, disparities in denominator population estimates across studies may further skew seroprevalence estimates [3].

### Risk factors associated with EIA transmission

Risk factor analysis highlighted that unauthorized animal movements, particularly across borders or between properties, are major drivers of disease dissemination. The introduction of an infected equine into a naïve population – especially in the absence of valid Animal Transit Guide documentation – represents the most critical determinant of within-herd transmission [48]. Additional risk factors include the use of mules in agricultural labor (e.g., cocoa farming), older animal age (>10 years), and mixed-breed status [49]. Uniform breeding and health practices across properties, coupled with limited enforcement of veterinary controls, further contribute to regional outbreaks.

### Diagnostic approaches and performance variability

Subgroup meta-analysis confirmed the widespread use of AGID, ELISA, PCR, and combined assays for EIAV diagnosis. AGID, which targets antibodies against the p26 core protein, remains a globally employed and cost-effective diagnostic method [50, 51]. Historically derived from the spleens of acutely infected horses, AGID antigens have now transitioned to recombinant or cell culture-based production [52]. Despite its specificity, AGID’s sensitivity may be compromised during early or low-titer infections.

ELISA-based methods, targeting p26 and occasionally gp45 antigens offer improved sensitivity and are widely used for screening. Non-commercial or modified ELISA approaches may be employed in resource-limited settings, particularly where standardized kits are unavailable [53, 54]. A validation study demonstrated high diagnostic performance of ELISA when confirmed by AGID, with sensitivity and specificity reaching 91.4% and 97.4%, respectively [55].

Recent innovations include B-cell epitope-based blocking ELISA and immunochromatographic assays such as colloidal gold-based test strips [56, 57]. These platforms offer rapid and field-deployable options for antibody detection and are especially useful for point-of-care screening. Nonetheless, competitive ELISAs may face limitations in detecting diverse EIAV strains due to epitope variability, necessitating monoclonal antibodies that target conserved regions [58, 59].

## CONCLUSION

This systematic review and meta-analysis provide the most comprehensive synthesis to date of the global prevalence and epidemiological patterns of EIA in Equidae. Based on 29 eligible studies comprising 27,909 samples from 16 countries, the pooled global prevalence was estimated at 20.97% (95% CI: 11.08–30.85), with substantial heterogeneity (I^2^ = 99.3%). Horses demonstrated the highest species-specific susceptibility (25.40%), and South America emerged as the region with the highest regional prevalence (27.21%). Seasonal patterns, particularly during summer, and risk factors such as unauthorized animal movement, mixed-breed populations, and inadequate vector control were identified as key contributors to disease spread. AGID remains the most widely employed diagnostic tool, though ELISA and PCR offer enhanced sensitivity in early or subclinical infections.

One of the key strengths of this study lies in its rigorous adherence to PRISMA guidelines, comprehensive database coverage, and stratified meta-analytic approach, which enabled subgroup and meta-regression analyses by continent, host species, diagnostic method, and time period. The use of geospatial mapping further enhances the contextual understanding of EIA surveillance gaps and endemic risks.

The practical implications of these findings are significant. The data underscore the urgent need for expanded serological surveillance, particularly in underreported and high-risk regions. National and international veterinary authorities should prioritize the implementation of standardized diagnostic protocols, enforce quarantine measures for seropositive equines, and control illegal cross-border animal movement to limit disease transmission. The integration of seasonal vector control strategies into national EIA management plans may also enhance the effectiveness of current prevention efforts.

However, the study has certain limitations. The underrepresentation of studies from Africa and Oceania, the exclusion of non-English publications, and the variability in diagnostic techniques and sample sizes across studies may introduce bias in prevalence estimates. In addition, the absence of data on climate, vector density, and animal movement patterns in many reports limits the ability to fully model transmission dynamics.

Future research should focus on expanding molecular epidemiology and genomic surveillance of EIAV strains, developing universally competitive ELISA platforms targeting conserved epitopes, and integrating environmental and socioeconomic factors into predictive models. Longitudinal surveillance studies across diverse climatic zones and equine populations will be critical to better understand the virus’s transmission ecology.

In conclusion, while the global burden of EIA remains unevenly distributed, this study reinforces the need for coordinated, evidence-based control efforts. Enhanced surveillance, diagnostic refinement, and cross-border cooperation are essential to mitigate the ongoing risk posed by this neglected yet economically significant equine disease.

## Data Availability

All the generated data are included in the manuscript.

## AUTHORS’ CONTRIBUTIONS

MTEP: Conceptualized and designed the study methodology. LWF, FF, APW, and MTEP: Curated and extracted the data. FF, MTEP, and HÇ: Analyzed the data, validated, and visualized the figures and tables. LWF, HÇ, and MTEP: Drafted and revised the manuscript. All authors have read and approved the final manuscript.
